# Public perception of isolation, quarantine, social distancing and community containment during COVID-19 pandemic

**DOI:** 10.1186/s12889-022-12970-y

**Published:** 2022-03-17

**Authors:** Tahreem Hussain, Nida Jawed, Saba Mughal, Kashif Shafique

**Affiliations:** 1 School of Public Health, Dow University of Health Sciences, OJHA Campus, Suparco Road, Karachi, 75270 Pakistan; 2 Institute of Health & Wellbeing, Public Health, University of Glasgow, 1-Lilybank Gardens, Glasgow, G12 8RZ UK

**Keywords:** COVID-19, Knowledge, Perception, Quarantine, Isolation, Community containment, Social distancing

## Abstract

**Background:**

Effective strategies of prevention have been and can aid in reducing and overcoming contagious diseases including COVID-19, still there is dearth of knowledge regarding general public awareness and perception. The current study aims to determine the existing knowledge and perception of people living in Karachi about isolation, quarantine, social distancing and community containment.

**Methods:**

This cross-sectional online survey was conducted during the months of March and April 2020. The study included men and women of age 18 years and above quarantined during COVID-19. Convenience sampling followed by snowball sampling technique was used. An online structured questionnaire was developed using Google Form. It included questions on socio demographic information, public knowledge and perception about isolation, quarantine, social distancing and community containment. Chi-square test was used for categorical variables and *p* value of < 0.05 was considered statistically significant.

**Results:**

A total of 548 participants were involved in this survey, 34% (*n* = 184) males with a mean age of 28 ± 10 years ranging from 18 to 75 years. The major source of hearing about COVID-19 was social media (72%, *n* = 393). Overall knowledge scores revealed that 27% (*n* = 146) participants had excellent knowledge about symptoms and prevention of the COVID-19. Excellent knowledge of quarantining, isolation and community containment and social distancing was 38% among participants. Participants who had good and excellent knowledge were more likely to have positive perception of isolation (*p*-value < 0.001). Majority participants (89%, *n* = 487) felt isolation may or may not be against human rights and this perception was found significantly associated with moderate to excellent knowledge about community mitigation measures (*p*-value = 0.009).

**Conclusion:**

This study concludes that optimal public knowledge and perception related to certain aspects of isolation, quarantine, social distancing and community containment exists however knowledge gaps and misperceptions prevail that need to be addressed.

**Supplementary Information:**

The online version contains supplementary material available at 10.1186/s12889-022-12970-y.

## Background

Globally, emerging and reemerging diseases are a serious threat to public health [1, 2]. The mechanism of the spread of COVID-19 is similar to any other common cold or influenza viruses including face to face contact with a sneeze or cough, or from interaction with secretions of people who are infected like hand shake [3]. Studies on SARS and the 2009 influenza pandemic proved that in the absence of vaccines and antivirals, traditional public health measures including isolation, quarantining and social distancing play a pivotal role in eradicating the diseases completely [4, 5]. COVID-19 vaccine has been developed and safe distribution has initiated. Yet the effects associated with COVID-19 vaccination are unprecedented [6, 7]. Therefore, the only way to curb the infection and control the COVID-19 pandemic is by relying on the old style public health measures [4, 8]. The basic idea behind these public health interventions is to prevent and limit the person-to-person close physical interaction thereby interrupting the disease transmission. Existing data revealed that isolation, quarantining, social distancing and community containment showed positive results in combating the spread of COVID-19 in China. Therefore globally, developed and developing countries followed the same classical tools to combat the frightening COVID-19 [9, 10].

Isolation of cases and contacts has long been a strategy in the fight against communicable diseases [11]. It can be defined as the separation of ill persons with contagious diseases from non-infected persons to protect non-infected persons, and usually occurs in hospital settings. Isolation of patients is particularly effective in interrupting transmission if early detection is possible before explicit viral spreading [4].

‘Quarantine’ is defined as the restriction of movement, upon an individual or a group of people to restrain them at a designated facility or in their homes. It is basically separation of healthy people from those who may have been exposed to the pathogen. The whole concept is to keep a track of people who may develop symptoms and ensure early detection of cases. It is one of the oldest and most effective tools of controlling infectious disease outbreaks [4]. Quarantine is voluntary and can include discouraging mass gatherings, closure of workplaces and educational institutes [11].

The third intervention “Social Distancing” also intends to reduce the spread of infection by reducing the interaction between the people of a community. As the mechanism of virus spread includes droplet inhalation the concept is to keep a distance so that people who may be infectious but are not identified cannot cause further transmission of disease to other people of the community [4].

Furthermore, ‘Communitywide Containment”, as the name suggests this intervention is applied to the whole community, city, or province. This intervention is restricting personal interaction at maximum. Such an intervention is more ethically complex as it involves a larger population [4]. Although these modes of prevention have been and can aid in reducing and overcoming the contagious diseases, still scarce is known about the general public awareness and perception related to them.

The rationale of the study was to assess the knowledge and perception regarding COVID-19 among general population residing in Karachi, Pakistan. Huge body of literature on knowledge and perception on COVID-19 is available but limited data exists on public knowledge and perception about classical preventive strategies in coping and eradicating infectious diseases such as COVID-19 in developing countries particularly in Pakistan. Furthermore, Isolation, quarantine and social distancing not only impacts people’s daily routine but also affects them mentally, physically, financially and socially. Therefore, it is crucial that people have correct knowledge and positive perception about these public health interventions in overcoming COVID-19. Only then they will show willingness to follow these preventive measures and adapt them easily. This will not only help in combating COVID-19 but will also aid in preventing and eradicating other infectious diseases. The current study will assess existing knowledge of population and will provide baseline data for planning effective interventions and initiatives to propagate these classical approaches for the management of COVID-19.

## Methods

### Aim, design and setting

The aim of this study was to assess existing public perception and knowledge about isolation, quarantine, social distancing and community containment among Karachi residents. A cross-sectional, observational study was conducted among the general public of Karachi, Pakistan during the outbreak of COVID-19. Because it was not feasible to conduct a community-based physical survey, the research team planned to do it online. Sample size was calculated using 4% margin of error and 95% confidence level, and the proportion of knowledge and awareness (65.6%) among young adults of Karachi. The sample size came out to be 542 [12]. Initially, a total of 569 participants were involved in this survey. After excluding missing values, final analysis was carried out on 548 participants. Participants aged 18 years and above were selected using convenience sampling technique followed by snowball sampling. The study proposal was reviewed and approved by the Institutional Review Board (IRB-1682/DUHS/Approval/2020) at Dow University of Health Sciences, Karachi. The online consent form was attached at the beginning of the questionnaire and only those individuals who agreed were able to participate in the study.

An online structured questionnaire was developed using Google Forms after reviewing the relevant available literature and published resources on isolation, quarantine, social distancing and community containment. After the questionnaire was developed, it was shared with the participants by a URL link through Email, WhatsApp, and other social media platforms like Facebook. The survey took approximately 12–15 min to complete. The participants of the study were motivated and encouraged to share the survey form with people in their contact information. Therefore, the link was shared to people other than the first point of contact person and so on. The participants who only consented to be a part of the survey were then able to proceed with the actual survey questionnaire.

Being an online study, only participants having an internet access were included in the survey. Male and female participants with age 18 years or more and residing in Karachi during COVID-19 lock down and were able to understand English were included in the study. However, individuals, under 18 years and not able to comprehend English language were excluded from the study. Data collection was initiated on March 30th, 2020 at 12:00 pm PKT and closed on April 30th, 2020, 12:00 am PKT. An online self-reported 26 item closed ended questionnaire was developed by the investigators containing 3 sections including general and socio demographic information, public knowledge and perception about isolation, quarantine, social distancing and community containment.

### Assessment instruments and variables

#### General and Sociodemographic information

Participant’s information including age, gender, education, ethnicity, area of residence, employment status and information regarding COVID-19 was recorded. Furthermore, in this section 7 questions regarding general information on COVID − 19 and its mitigation strategies were asked from the participants.

#### Knowledge

In the questionnaire 11 items related to knowledge were asked from the participants. The section on knowledge was assessed through multiple choice questions. From a total 7 items, 4 questions assessed knowledge regarding the COVID-19, its signs and symptoms and prevention. Three questions were asked on the knowledge regarding the mitigation strategies including: isolation, quarantine, and community containment. In addition, 4 items were asked about the knowledge on social distancing.

#### Perception

A total of 7 items were used to assess participant’s perception about community mitigation strategies including isolation, quarantine, social distancing and community containment.

For study questionnaire reliability we computed the value coefficient omega for knowledge and perception measures which was 0.68 indicating level of acceptance for the survey [13].

### Statistical analysis

The analysis was conducted using Statistical Package for Social Sciences (SPSS) version 16.0. Descriptive statistics were reported as percentage and frequency for all categorical variables and as mean ± standard deviation for quantitative variables. Knowledge scores about symptoms and prevention of the COVID-19, quarantining, isolation, community containment and social distancing were generated using their respective questions. Every correct response was scored 1 and wrong response was scored 0. After summing, final knowledge scores were categorized on the basis of quartiles into four groups and labeled as poor, moderate, good and excellent from lowest quartile to highest quartile. Cutoff values were (Q_1_ = 22, Q_2_ = 23, Q_3_ = 25; Poor = 17–21, Moderate = 22, Good = 23–24, Excellent = 25–27), (Q_1_ = 2, Q_2_ = 3, Q_3_ = 4; Poor = 0–1, Moderate = 2, Good = 3, Excellent = 4–5) and (Q_1_ = 12, Q_2_ = 14, Q_3_ = 15; Poor = 6–11, Moderate = 12–13, Good = 14, Excellent = 15–17) for knowledge scores about symptoms and prevention of the disease, about quarantining, isolation, community containment and about social distancing, respectively. Chi-square test was used to determine the relationship between the outcome variables related to participants’ perceptions about actions being taken by the government during COVID-19 pandemic and all the independent variables of knowledge. All test results having *p*-values less than or equal to alpha (0.05) were considered statistically significant.

## Results

A total of 569 participants were involved in this survey, after excluding missing values, final analysis was carried out on 548 participants. Among all the individuals, 34% (*n* = 184) were males and 66% (*n* = 364) were females. Mean age in years with ±SD was 28 ± 10 ranging from 18 to 75 years. Majority of the participants (75%, *n* = 410) were graduate (Table 1). For the participants, the major source of hearing about COVID-19 pandemic for the first time was social media (72%, *n* = 393) followed by television (54%, *n* = 297). Around 12 % (*n* = 65) participants had COVID-19 patients in their area whereas only 2 % (*n* = 11) had COVID-19 patients in their family. Most participants reported fever (95%, *n* = 518), dry cough (89%, *n* = 485) and shortness of breath (87%, *n* = 476) as one of the common symptoms of the disease. Majority (88%, *n* = 482) also think that COVID-19 can be prevented. Social distancing (96%, *n* = 526), washing hands frequently with soap (94%, *n* = 515) and use of hand sanitizers (92%, *n* = 503) were reported with high proportions as one of the ways of prevention, whereas 70% (*n* = 384) participants also considered that increasing intake of vitamin C can help preventing the disease (Table 2).

**Table 1:** Socio-demographic characteristics of the respondents (*n* = 548)

Characteristics	***n***	%
**Age (years)**		
< 25	291	53.1
≥ 25	257	46.9
**Gender**		
Female	364	66.4
Male	184	33.6
**Level of education**		
Intermediate or below	70	12.8
Graduate	410	74.8
Post graduate	68	12.4
**Ethnicity**		
Sindhi	141	25.7
Punjabi	69	12.6
Baluchi	3	0.5
Pathan	25	4.6
Other	310	56.6
**Employment status**		
Unemployed	215	39.2
Employed	214	39.1
Self-employed	41	7.5
Other	73	13.3
Retired	5	0.9
**Area of residence- Districts**		
Central	96	17.5
East	103	18.8
South	83	15.1
West	100	18.3
Korangi	81	14.8
Malir	85	15.5

**Table 2:** Participants’ responses regarding symptoms & prevention of COVID-19 pandemic (*n* = 548)

Response	***n***	%
**From whom/where did you hear first about COVID-19?**^**a**^		
Family	102	18.6
Friends	92	16.8
At religious setting	6	1.1
Television	297	54.2
At office	49	8.9
Social media	393	71.7
Radio	7	1.3
Newspaper	53	9.7
**Does anyone in your area have COVID-19?**		
Yes	65	11.9
No	297	54.2
Maybe	186	33.9
**Does anyone in your family have COVID-19?**		
Yes	11	2.0
No	523	95.4
Maybe	14	2.6
**What are the signs and symptoms of COVID-19?**^**a**^		
Fever	518	94.5
Flu	372	67.9
Diarrhea	118	21.5
Runny nose	198	36.1
Skin rash	10	1.8
Dry cough	485	88.5
Joint pain	133	24.3
Vomiting	56	10.2
Shortness of breath	476	86.9
Nose bleed	10	1.8
Severe weakness	205	37.4
High blood pressure	14	2.6
Red eyes	52	9.5
**Do you think diet plays a role in the prevention in COVID-19?**		
Yes	371	67.7
No	70	12.8
Maybe	107	19.5
**Can COVID-19 be prevented?**		
Yes	482	88.0
No	9	1.6
Maybe	57	10.4
**If yes, how can you prevent it?**^**a**^		
Using mosquito repellant	17	3.1
Avoiding meat, poultry & eggs	37	6.8
Social distancing	526	96.0
Removing stagnant water	35	6.4
Quarantining	485	88.5
Increasing vitamin C intake	384	70.1
Using hand sanitizers	503	91.8
Wearing face masks	478	87.2
Drinking clean water	251	45.8
Washing hands frequently with soap	515	94.0
Isolation	474	86.5
Hygiene	414	75.5

^a^Multiple options were selected

Overall knowledge scores revealed that 27% (*n* = 146) participants had excellent knowledge about symptoms and prevention of the disease. Overall knowledge of quarantining, isolation and community containment was poor among 7.7% (*n* = 42) of the participants whereas excellent among 38% (*n* = 206) of the participants. Around 38% (*n* = 207) of the participants had excellent knowledge about social distancing (Table 3).

**Table 3:** Participants’ knowledge about symptoms and prevention, quarantining, isolation, community containment and social distancing during COVID-19 pandemic (*n* = 548)

Knowledge	***n***	%
**Knowledge about symptoms & prevention**		
Poor	107	19.5
Moderate	82	15.0
Good	213	38.9
Excellent	146	26.6
**Knowledge about quarantining, isolation and community containment**		
Poor	42	7.7
Moderate	133	24.3
Good	167	30.5
Excellent	206	37.6
**Knowledge about social distancing**		
Poor	102	18.6
Moderate	118	21.5
Good	121	22.1
Excellent	207	37.8

Demographic differences in knowledge were assessed and reported in Table 4. It was noted that proportion of excellent knowledge about quarantining and isolation was high in those respondents, who aged less than 25 years (57%, *n* = 118), were females (74%, *n* = 152), were graduate (81%, *n* = 166) and employed (63%, *n* = 130) as compared to those who were more than 25 years old, males, intermediate or post graduate and unemployed or retired. Age (*p*-value < 0.001), gender (*p*-value = 0.005), education (*p*-value < 0.001) and employment status (*p*-value = 0.047) were significantly associated with the knowledge about quarantining, isolation and community containment. Level of education was also positively associated with the knowledge about social distancing (*p*-value = 0.042).

**Table 4:** Socio-demographic characteristics of the participants with their knowledge about COVID-19 pandemic

Characteristics	Knowledge about symptoms & prevention				Knowledge about quarantining, isolation and community containment				Knowledge about social distancing			
	Poor	Moderate	Good	Excellent	Poor	Moderate	Good	Excellent	Poor	Moderate	Good	Excellent
**Age (years)**												
< 25	53 (49.5)	43 (52.4)	119 (55.9)	76 (52.1)	22 (52.4)	84 (63.2)	67 (40.1)	118 (57.3)	51 (50.0)	57 (48.3)	69 (57.0)	114 (55.1)
≥ 25	54 (50.5)	39 (47.6)	94 (44.1)	70 (47.9)	20 (47.6)	49 (36.8)	100 (59.9)	88 (42.7)	51 (50.0)	61 (51.7)	52 (43.0)	93 (44.9)
**V,** ***p*-value**	0.048, 0.734				0.182, < 0.001				0.068, 0.465			
**Gender**												
Female	65 (60.7)	60 (73.2)	150 (70.4)	89 (61.0)	24 (57.1)	92 (69.2)	96 (57.5)	152 (73.8)	62 (60.8)	81 (68.6)	79 (65.3)	142 (68.6)
Male	42 (39.3)	22 (26.8)	63 (29.6)	57 (39.0)	18 (42.9)	41 (30.8)	71 (42.5)	54 (26.2)	40 (39.2)	37 (31.4)	42 (34.7)	65 (31.4)
**V,** ***p*-value**	0.111, 0.082				0.154, 0.005				0.064, 0.527			
**Level of education**												
Intermediate or below	18 (16.8)	9 (11.0)	28 (13.1)	15 (10.3)	8 (19.0)	31 (23.3)	14 (8.4)	17 (8.3)	15 (14.7)	13 (11.0)	20 (16.5)	22 (10.6)
Graduate	76 (71.0)	62 (75.6)	163 (76.5)	109 (74.7)	31 (73.8)	90 (67.7)	123 (73.7)	166 (80.6)	82 (80.4)	93 (78.8)	86 (71.1)	149 (72.0)
Post graduate	13 (12.1)	11 (13.4)	22 (10.3)	22 (15.1)	3 (7.1)	12 (9.0)	30 (18.0)	23 (11.2)	5 (4.9)	12 (10.2)	15 (12.4)	36 (17.4)
**V,** ***p*-value**	0.062, 0.641				0.157, < 0.001				0.109, 0.042			
**Employment status**												
Unemployed/ Retired	38 (35.5)	35 (42.7)	85 (39.9)	62 (42.5)	17 (40.5)	67 (50.4)	60 (35.9)	76 (36.9)	37 (36.3)	45 (38.1)	53 (43.8)	85 (41.1)
Employed	69 (64.5)	47 (57.3)	128 (60.1)	84 (57.5)	25 (59.5)	66 (49.6)	107 (64.1)	130 (63.1)	65 (63.7)	73 (61.9)	68 (56.2)	122 (58.9)
**V,** ***p*-value**	0.052, 0.681				0.120, 0.047				0.054, 0.664			

*n* (%) are reported. V = Effect size by Cramer’s V coefficient

*p-values were calculated using the chi-squared test

Of the total, 64% (*n* = 349) respondents thought isolating a large population inside their houses forcefully during this pandemic is ethically sound. Knowledge about quarantining, isolation and community containment was positively associated with the perception of isolating a large population as those participants who had good and excellent knowledge were more likely to have positive perception of isolation (*p*-value < 0.001). Participants who felt isolating people forcefully in their home is not or maybe against human rights were 89% (*n* = 487) and this perception was found significantly associated with moderate to excellent knowledge about quarantining, isolation and community containment (*p*-value = 0.009) (Table 5).

**Table 5:** Respondents’ perceptions over government’s actions related to human rights during COVID-19 pandemic

Perception	***n*** (%)	***n*** (%)	V, ***p***-value^*^
**Do you think to isolate large population inside their houses forcefully during this COVID-19 is ethically sound?**			
	**Yes (*n*** **= 349)**	**No/ Maybe (*n*** **= 199)**	
Knowledge about symptoms & prevention			
Poor	65 (60.7)	42 (39.3)	0.133, 0.022
Moderate	45 (54.9)	37 (45.1)	
Good	152 (71.4)	61 (28.6)	
Excellent	87 (59.6)	59 (40.4)	
Knowledge about quarantining, isolation and community containment			
Poor	13 (31.0)	29 (69.0)	0.197, < 0.001
Moderate	87 (65.4)	46 (34.6)	
Good	110 (65.9)	57 (34.1)	
Excellent	139 (67.5)	67 (32.5)	
Knowledge about social distancing			
Poor	59 (57.8)	43 (42.2)	0.093, 0.193
Moderate	72 (61.0)	46 (39.0)	
Good	86 (71.1)	35 (28.9)	
Excellent	132 (63.8)	75 (36.2)	
**Do you feel isolating people forcefully in their home is against human rights?**			
	**Yes (*n*** **= 61)**	**No/ Maybe (*n*** **= 487)**	
Knowledge about symptoms & prevention			
Poor	16 (15.0)	91 (85.0)	0.067, 0.477
Moderate	7 (8.5)	75 (91.5)	
Good	24 (11.3)	189 (88.7)	
Excellent	14 (9.6)	132 (90.4)	
Knowledge about quarantining, isolation and community containment			
Poor	11 (26.2)	31 (73.8)	0.145, 0.009
Moderate	12 (9.0)	121 (91.0)	
Good	20 (12.0)	147 (88.0)	
Excellent	18 (8.7)	188 (91.3)	
Knowledge about social distancing			
Poor	17 (16.7)	85 (83.3)	0.094, 0.187
Moderate	9 (7.6)	109 (92.4)	
Good	12 (9.9)	109 (90.1)	
Excellent	23 (11.1)	184 (88.9)	

V = Effect size by Cramer’s V coefficient

*p-values were calculated using the chi-squared test

More than half of participants (56%, *n* = 306) felt that the closing of religious places is religiously sound. This perception was positively associated with higher knowledge about quarantining, isolation and community containment (*p*-value = 0.015). Seventy four percent of the participants (*n* = 405) thought that people are not or maybe obeying the government’s order of restraining from gatherings. Respondents with moderate and good knowledge about social distancing were more likely to support this perception (81 and 80%) compared with those who had poor knowledge about social distancing (60%, *p*-value 0.001) (Table 6).

**Table 6:** Respondents’ perceptions over government’s actions related to religion during COVID-19 pandemic

Perception	***n*** (%)	***n*** (%)	V, ***p***-value^*^
**Do you feel the closing of religious places (Masjid/church/mandir, etc) is religiously sound?**			
	**Yes (*n*** **= 306)**	**No/ Maybe (*n*** **= 242)**	
Knowledge about symptoms & prevention			
Poor	60 (56.1)	47 (43.9)	0.064, 0.520
Moderate	41 (50.0)	41 (50.0)	
Good	126 (59.2)	87 (40.8)	
Excellent	79 (54.1)	67 (45.9)	
Knowledge about quarantining, isolation and community containment			
Poor	14 (33.3)	28 (66.7)	0.138, 0.015
Moderate	74 (55.6)	59 (44.4)	
Good	102 (61.1)	65 (38.9)	
Excellent	116 (56.3)	90 (43.7)	
Knowledge about social distancing			
Poor	53 (52.0)	49 (48.0)	0.067, 0.485
Moderate	61 (51.7)	57 (48.3)	
Good	70 (57.9)	51 (42.1)	
Excellent	122 (58.9)	85 (41.1)	
**Do you think to restrict people from visiting their religious places (Masjid/Chruch/Mandir) will cause agitation (anxiety/tension) among people?**			
	**Yes (*n*** **= 282)**	**No/ Maybe (*n*** **= 266)**	
Knowledge about symptoms & prevention			
Poor	63 (58.9)	44 (41.1)	0.117, 0.057
Moderate	48 (58.5)	34 (41.5)	
Good	107 (50.2)	106 (49.8)	
Excellent	64 (43.8)	82 (56.2)	
Knowledge about quarantining, isolation and community containment			
Poor	20 (47.6)	22 (52.4)	0.042, 0.814
Moderate	68 (51.1)	65 (48.9)	
Good	83 (49.7)	84 (50.3)	
Excellent	111 (53.9)	95 (46.1)	
Knowledge about social distancing			
Poor	50 (49.0)	52 (51.0)	0.092, 0.204
Moderate	65 (55.1)	53 (44.9)	
Good	70 (57.9)	51 (42.1)	
Excellent	97 (46.9)	110 (53.1)	
**Do you think people are obeying the government’s order of restraining from gatherings?**			
	**Yes (*n*** **= 143)**	**No/ Maybe (*n*** **= 405)**	
Knowledge about symptoms & prevention			
Poor	24 (22.4)	83 (77.6)	0.069, 0.456
Moderate	21 (25.6)	61 (74.4)	
Good	53 (24.9)	160 (75.1)	
Excellent	45 (30.8)	101 (69.2)	
Knowledge about quarantining, isolation and community containment			
Poor	10 (23.8)	32 (76.2)	0.067, 0.482
Moderate	32 (24.1)	101 (75.9)	
Good	51 (30.5)	116 (69.5)	
Excellent	50 (24.3)	156 (75.7)	
Knowledge about social distancing			
Poor	41 (40.2)	61 (59.8)	0.169, 0.001
Moderate	23 (19.5)	95 (80.5)	
Good	24 (19.8)	97 (80.2)	
Excellent	55 (26.6)	152 (73.4)	

V = Effect size by Cramer’s V coefficient

^*^p-values were calculated using the chi-squared test

## Discussion

The current study was conducted during the initial phase of COVID-19 outbreak with the aim of assessing public perception and knowledge about isolation, quarantine, social distancing and community containment. According to our study results, the majority of the participants lack adequate knowledge regarding non-pharmaceutical interventions. Only one third of the study population had excellent knowledge regarding quarantining, isolation, community containment and social distancing.

During the course of this study more researches have been published on similar topic. Recently published study in Netherlands, Germany, Italy, USA and China highlighted the public perspectives on protective measures during the COVID-19 pandemic [14]. It is worth mentioning that the majority of our study participants considered COVID-19 as preventable. Good Hygiene is considered to be one of the most effective preventive measures of communicable diseases. Handwashing is an important basic personal hygiene practice that can help in reducing transmission of COVID-19 [15]. Our study findings also revealed excellent knowledge scores regarding its preventive measures. Almost all the participants had excellent knowledge regarding social distancing, washing hands frequently with soap and use of hand sanitizers whereas three quarter of the population considered increased vitamin C intake as an effective measure in COVID-19 prevention. Furthermore, most of the study participants indicated fever, dry cough and shortness of breath as common signs and symptoms of the disease. Another interesting study finding revealed that almost three fourth of our population, first heard about the COVID-19 through social media followed by television which accounted for more than half of the population.

Although in the current study no gender differences were found unlike other studies where there was significant differences found in knowledge and perception in gender [8, 16].

The majority of participants had moderate to excellent knowledge about COVID-19 and perceived forceful isolation as not against human rights. Likewise, two third of the population considered house isolation by force during the pandemic situation as ethically sound. In addition, to this a positive perception about isolation was observed only among population with good to excellent knowledge. According to our study findings more than half of our respondents considered closure of religious places as religiously sound. This positive perception was found in participants having higher knowledge about quarantining, isolation and community containment. Similarly, three fourth of the participants had mixed opinion regarding following the government’s order of restraining from public gatherings. This perception was observed by most of the participants with moderate and good knowledge about social distancing.

The current study findings related to knowledge about COVID-19 and its public health strategies are parallel to previous studies conducted in developed and developing countries [2, 17–21]. During this pandemic, social media played a pivotal role in disseminating both information and misinformationAt one end, it helped in creating awareness and knowledge among the general population. While on the other hand, it rapidly spread disinformation and rumors among the masses [15]. Similar findings were found in Chinese and Iranian studies conducted online [1, 20, 22]. Therefore, this medium of communication can prove effective for disseminating valid and reliable knowledge that would help the general public to clear misconceptions related to this pandemic. This high percentage might be due to extensive use of social media and television. Many studies conducted on COVID-19 observed similar findings [18, 20, 22, 23]. A European study highlighted similar importance of social media e.g. Facebook, Twitter platform as a source of information in almost half of the population [14].

Similar to other Muslim countries, restriction on religious activities and public gatherings during COVID-19 outbreak has been a controversial topic in Pakistan as well. Government of Pakistan restricted congregational activities including closure of prayers, sermons, milads, shrines, and Friday (Jummah) prayers [23–26]. From the study population, majority of participants perceived that restraining from religious gatherings would prevent the spread of infection. Therefore, these steps are in line with other Muslim countries’ response to COVID-19 which focused on isolation, quarantine and community containment.

To the best of our knowledge this is the first study to discuss public perception and knowledge about classical preventive strategies including isolation, quarantine, social distancing and community containment during this COVID-19 outbreak. The findings of this study will help to design public health initiatives, programs and strategies to plan subsequent interventions in the preparedness during pandemic situations. As highlighted in the study social media plays a pivotal role in spreading information about COVID-19. Therefore, this medium of communication can prove effective for disseminating valid and reliable knowledge that would help the general public to clear misconceptions related to this pandemic. Based on the findings of our study, the participant’s knowledge about public health strategies play a significant role in adhering to government’s policies and measures taken during the current pandemic. Moreover, this will help in mitigating the outbreak of the disease in its early phase and will draw more attention towards prevention of the disease rather than treatment. Thus through these community measures the people at risk will not only be able to prevent themselves but also seek medical advice at its earliest.

### Limitations

There are several limitations in our study. Firstly, data collection was confined to only one city of Pakistan. Hence the results of the study may not be very widely be generalizable. Secondly, internet access and a basic command over English language were pre-requisites to participation in the study, thus a large population of people who were illiterate were excluded from the study. The study data was limited to urban population of the city having access to internet facilities which can have an effect knowledge about the disease as compared to the rural population.

Data were deleted for incomplete information whereas an imputation procedure could be applied to fill in missing values.

Outcome variables were categorized into quartiles although analyses of continuous variables could have been done.

## Conclusion

This study concludes that optimal public knowledge and perception related to certain aspects of isolation, quarantine, social distancing and community containment exists however knowledge gaps and misperceptions also prevail that need to be addressed.

## Supplementary Information

**Additional file 1.**

## Data Availability

Dataset is available with the corresponding author and will be shared on request if necessary.
