# Supplementary material for: Public perception of isolation, quarantine, social distancing and community containment during COVID-19 pandemic
Source: BMC Public Health. 2022 Mar 17;22:528. doi: 10.1186/s12889-022-12970-y (PMC8931453; doi:10.1186/s12889-022-12970-y)
Supplement: Supplementary file 1 — Additional file 1. [file 12889_2022_12970_MOESM1_ESM.docx]

**Title Page**

**Public Perception of Isolation, Quarantine, Social Distancing and Community Containment during COVID-19 Pandemic**

*Tahreem Hussain^1^

Nida Jawed^1^

Saba Mughal^1^

Dr. Kashif Shafique^1,2^

^1^School of Public Health, Dow University of Health Sciences, OJHA Campus, Suparco Road, Karachi 75270, Pakistan

^2^Institute of Health & Wellbeing, Public Health, University of Glasgow,1-Lilybank Gardens, Glasgow G12 8RZ, United Kingdom

***Corresponding Author:**

Tahreem Hussain

E-mail: [tahreem.hussain@duhs.edu.pk](mailto:tahreem.hussain@duhs.edu.pk)

School of Public Health, Dow University of Health Sciences, OJHA Campus, Suparco Road, Karachi 75270, Pakistan^,^ Telephone: 923224638584.

**Consent:**

This questionnaire is designed to evaluate Public Perception of Isolation, Quarantine, Social Distancing and Community Containment during COVID-19 Pandemic:

This online survey is developed to identify the populations' perception on the Isolation, Quarantine, Social Distancing and Community Containment during COVID-19 pandemic and to see the awareness about these public health measures

Based on the data obtain from this self-reported survey, suggestions will be recommended on the potential behavioural determinants related to acceptance of quarantine option during this COVID-19 pandemic.

* There are no questions in the survey, which will cause you to reveal your identity or cause you any potential harm. Hence, your identity will remain anonymous. If you agree to participate, please fill the questions below.

#### Questionnaire:

#### Please fill the question

#### A. From whom/where did you hear first about COVID-19? (You can tick multiple options)

| 1. Family | 2. Friends | 3. At Religious    setting | 4. Television |
| --- | --- | --- | --- |
| 5. At office | 6. Social Media | 7. Radio | 8. Newspaper |

#### B. Does anyone in your area has COVID-19?

| 0. No | 1. Yes | 2. Maybe |
| --- | --- | --- |

#### C. Does anyone in your family has COVID-19?

| 0. No | 1. Yes | 2. Maybe |
| --- | --- | --- |

#### D. What are the signs and symptoms of COVID-19? (You can tick multiple options)

| 1.     Fever | 2.     Flu | 3.     Diarrhea | 4.     Runny Nose | 5.     Skin Rash |
| --- | --- | --- | --- | --- |
| 6.     Edema | 7.     Dry Cough | 8.      Anemia | 9.     Joint Pain | 10.  Vomiting |
| 11.  Shortness of breath | 12.  Nose bleed | 13.  Severe weakness | 14.  High Blood Pressure | 15.  Red eyes |

#### E. Do you think diet plays a role in the prevention in COVID-19?

| 0. No | 1. Yes | 2. Maybe |
| --- | --- | --- |

#### F. Can COVID-19 be prevented?

| 0. No | 1. Yes | 2. Maybe |
| --- | --- | --- |

#### G. If yes, how can you prevent it? (You can tick multiple options)

| 1.     Using mosquito repellant | 0.    No | 1.     Yes |
| --- | --- | --- |
| 2.     Avoiding meat, poultry & eggs | 0.    No | 1.   Yes |
| 3.     Social distancing | 0.    No | 1.   Yes |
| 4.     Removing stagnant water | 0.    No | 1.   Yes |
| 5.     Quarantining | 0.    No | 1.   Yes |
| 6.     Increasing vitamin C intake | 0.    No | 1.   Yes |
| 7.     Using hand sanitizers | 0.    No | 1.   Yes |
| 8.     Wearing face masks | 0.    No | 1.   Yes |
| 9.     Drinking clean water | 0.    No | 1.   Yes |
| 10.  Washing hands frequently with soap | 0.    No | 1.   Yes |
| 11.  Isolation | 0.    No | 1.   Yes |
| 12.  Hygiene | 0.    No | 1.   Yes |

#### H. Have you heard about the term “quarantine”?

| 0. No | 1. Yes | 2. Maybe |
| --- | --- | --- |

#### I.   What do you mean by quarantine?

| 1.     To separate healthy people from people with communicable disease |
| --- |
| 2.     To separate and restrict the movement of healthy people who are exposed to disease to see if they have no symptoms |
| 3.     Not sure |

#### J.  Have you heard about “isolation”?

| 0. No | 1. Yes | 2. Maybe |
| --- | --- | --- |

#### K. What do you mean by isolation?

| 1.     To separate healthy people from people with communicable disease |
| --- |
| 2.     To separate and restrict the movement of healthy people who are exposed to disease to see if they have no symptoms |
| 3.     Not sure |

#### L.     Have you heard about the term “community containment”?

| 0. No | 1. Yes | 2. Maybe |
| --- | --- | --- |

#### M.   What do you mean by community containment?

| 1. Maximum restriction of personal interaction at a massive level |
| --- |
| 2.Imprisonment of people in community for not following orders at the time of outbreak of disease |
| 3. Restricting only certain community members have personal interaction |
| 4. All of the above |
| 5. Not sure |

#### N. Have you heard about “social distancing”?

| 0. No | 1. Yes | 2. Maybe |
| --- | --- | --- |

#### O.    What do you mean by social distancing? (You can tick multiple options)

| 1. Distancing yourself from others to minimize the spread of contagious disease |
| --- |
| 2. Distancing from people in gatherings to reduce exposure to contagious disease |
| 3. Maintain distance from other people in public places during outbreak of disease |
| 4. All of the above |
| 5. Not sure |

#### P. Which of the following is an example of social distancing? (You can tick multiple options)

| 1.     Hand shaking | 0.    No | 1.     Yes |
| --- | --- | --- |
| 2.     Cancellation of PSL match | 0.    No | 1.     Yes |
| 3.     Working from home instead of office | 0.    No | 1.     Yes |
| 4.     Going for picnic on beach or park | 0.    No | 1.     Yes |
| 5.     Closing educational institutes | 0.    No | 1.     Yes |
| 6.     Cancelling any public events & gatherings | 0.    No | 1.     Yes |
| 7.     Inviting friends and family at home | 0.    No | 1.     Yes |
| 8.     Children playing street cricket | 0.    No | 1.     Yes |

#### Q. Why is social distancing important? (You can tick multiple options)

| 1.     Helps slow down spread of disease | 0.    No | 1.     Yes |
| --- | --- | --- |
| 2.     Helps identifying patients with disease | 0.    No | 1.     Yes |
| 3.     Helps health care providers to care for patients efficiently and effectively | 0.    No | 1.     Yes |
| 4.     Helps in the treatment of the patient at home | 0.    No | 1.     Yes |
| 5.     Helps to protect the healthy from infected people | 0.    No | 1.     Yes |

#### R. How much distance is considered to be appropriate in social distancing?

| 1. At least 6 feet | 2. At least 2 feet | 3. At least 4 feet | 4. Not sure |
| --- | --- | --- | --- |

#### S. Do you feel isolating yourself will prevent you from getting COVID-19 and spreading it to others?

| 0. No | 1. Yes | 2. Maybe |
| --- | --- | --- |

#### T. Do you feel quarantining yourself will prevent from getting COVID-19 and spreading it to others?

| 0. No | 1. Yes | 2. Maybe |
| --- | --- | --- |

####

#### U. Do you think isolating a large population inside their houses forcefully during this COVID-19 is ethically sound?

| 0. No | 1. Yes | 2. Maybe |
| --- | --- | --- |

#### V. Do you feel isolating people forcefully in their home is against human rights?

| 0. No | 1. Yes | 2. Maybe |
| --- | --- | --- |

#### W.  Do you feel the closing of religious places (Masjid/church/mandir, etc) is religiously sound?

| 0. No | 1. Yes | 2. Maybe |
| --- | --- | --- |

#### X. Do you think restricting people from visiting their religious places (Masjid/Church/Mandir) will cause agitation (anxiety/tension) among people?

| 0. No | 1. Yes | 2. Maybe |
| --- | --- | --- |

#### Y. Do you think people are obeying the government’s order of restraining from gatherings?

| 0. No | 1. Yes | 2. Maybe |
| --- | --- | --- |

#### Demographic Information

| X1. Age |  | | | | | |
| --- | --- | --- | --- | --- | --- | --- |
| X2. Gender | Male | | | | Female | |
| X3. Ethnicity | 1.     Sindhi | 2.     Baluchi | | 3.     Punjabi | | 4.     Pathan |
|  | 5.     Other_____________________ | | | | | |
| X4. Years of Education |  | | | | | |
| X5. Residence Area |  | | | | | |
| X6. Employment Status | 1.     Employed2.     Unemployed3.     Self-employed4.     Retired | | | | | |
|  |  |  |  |  | |  |
